# Association of *GSTP1* Ile105Val polymorphism with the risk of coronary heart disease: An updated meta-analysis

**DOI:** 10.1371/journal.pone.0254738

**Published:** 2021-07-22

**Authors:** Yadong Song, Xiaoli Liu, Cheng Luo, Liangkai Chen, Lin Gong, Hanbin Yu, Bin Wang, Ernan Liu, Huiqiong Xu, Jiansheng Liang

**Affiliations:** 1 Department of Disinfection and Pest Control, Wuhan Centers for Disease Prevention and Control, Wuhan, Hubei, China; 2 Wuhan Healthcare-associated Infection Management Quality Control Center, Wuhan, Hubei, China; 3 Department of Nutrition and Food Hygiene, Hubei Key Laboratory of Food Nutrition and Safety, Tongji Medical College, Hua Zhong University of Science and Technology, Wuhan, People’s Republic of China; 4 Ministry of Education Key Lab of Environment and Health, School of Public Health, Tongji Medical College, Wuhan, People’s Republic of China; Shanghai Jiao Tong University, CHINA

## Abstract

**Background:**

Numerous case-control studies have investigated the association between *GSTP1* Ile105Val polymorphism and CHD risk, but the results from published studies were inconclusive. The present meta-analysis was performed to derive a more precise estimation.

**Methods:**

PubMed, EMBASE, and Web of Science database searches were conducted to retrieve relevant articles.

**Results:**

Ultimately, 5,451 CHD cases and 5,561 controls from 15 studies were included. Pooled analysis did not yield any statistically significant association between *GSTP1* Ile105Val polymorphism and CHD risk for the overall population (Val vs. Ile: OR, 1.05; 95% CI, 0.93 to 1.18; Val/Val vs. Ile/Ile: OR, 1.09; 95% CI, 0.83 to 1.42; Val/Ile vs. Ile/Ile: OR, 1.09; 95% CI, 0.93 to 1.28; Val/Val vs. Val/Ile+Ile/Ile: OR, 1.04; 95% CI, 0.83 to 1.30; Val/Val+Val/Ile vs. Ile/Ile: OR, 1.14; 95% CI, 0.97 to 1.33). Subgroup analyses and sensitivity analyses indicated that *GSTP1* Ile105Val polymorphism was still not associated with an increased risk of CHD. After excluding studies detected by Galbraith plots as major sources of heterogeneity, these relationships were still not significant.

**Conclusions:**

The overall results did not reveal a major role of the *GSTP1* Ile105Val polymorphism in modulating CHD risk. Well-designed studies with large sample sizes are needed to validate our findings and explore the possible gene-gene or gene-environment interactions.

## Introduction

Atherosclerosis is a major cause of coronary heart disease (CHD), a major public health problem, and a leading cause of morbidity and mortality in the world [1,2]. It is well known that the complex interaction of environmental and predisposing genetic risk factors plays a crucial role in the underlying pathophysiology of CHD. Multiple traditional risk factors lead to CHD development, including age, a high-fat diet, smoking, alcohol, diabetes mellitus, hyperlipidemia, hypertension, and so on.

Oxidative stress, an imbalance between antioxidant defenses and free radical generation, was implicated as potential pathophysiological mechanisms behind the pathogenesis and progression of CHD [3]. DNA damage, inflammation, smooth muscle cell proliferation, and lipid peroxidation, which are caused by increased production of reactive oxygen species (ROS), can result in atherosclerosis and, hence, CHD [4]. Furthermore, DNA adducts have been detected in patients with severe CHD [5] and atherosclerotic plaques [6]. DNA adducts were considered to be related to atherogenic risk factors including old age, alcohol drinking status, smoking status, oxidative DNA damage, triglycerides, cholesterol, and arterial pressure. Besides, DNA adducts have been thought to be risk factors for reducing the capacity of antioxidants [7].

Glutathione S-transferases (GSTs) are a superfamily of phase II detoxification enzymes that convert various reactive metabolites (endogenous and exogenous products of oxidative stress) to more water-soluble and less harmful forms by conjugating them with glutathione [8]. Therefore, GSTs play vital roles in protecting the cell against oxidative stress. Besides, GSTs can protect DNA from genotoxic damage by inhibiting the formation of DNA adducts [9]. Human GSTs are composed of eight major groups including GSTM (mu), GSTT (theta), GSTP (pi), GSTA (alpha), GSTK (kapa), GSTO (omega), GSTS (sigma) and GSTZ (zeta) [10]. The glutathione S-transferase P1 (*GSTP1*) gene is 2.8 KB long, which is located on the long arm of chromosome 11 (11q13.3). The *GSTP1* Ile105Val polymorphism is a single nucleotide polymorphism (SNP) located in exon 5 which encodes an isoleucine (Ile) to valine (Val) exchange at codon 105. Individuals with *GSTP1* protein containing Val but not Ile have significantly reduced enzyme activity and affinity for electrophilic substrates [11]. Thus, decreased detoxification ability of lower enzymatic activity may increase the risk of CHD.

A number of case-control studies [4,7,8,12–23] have investigated the relationship between *GSTP1* Ile105Val polymorphism with the risk of CHD, with equivocal conclusions. Recently, one meta-analysis [24] revealed that a significant association exists between *GSTP1* null genotype and CHD, and the other one [25] suggested that *GSTP1* null genotype could impact individual susceptibility to atherosclerotic cardiovascular diseases. However, numerous relevant publications [4,19,22,23] did not appear in their meta-analyses. Moreover, their meta-analyses did not evaluate the strength of the association between *GSTP1* Ile105Val polymorphism and CHD risk in the specific genetic models, dominant model, homozygote model, heterozygote model, recessive model, and an allele comparison, respectively. Therefore, we performed an updated meta-analysis to establish a comprehensive picture of the Ile105Val polymorphism of the *GSTP1* and the risk of CHD.

## Materials and methods

### Identification and eligibility of relevant studies

Basing on the Preferred Reporting Items for Systematic Reviews and Meta-Analyses (PRISMA), we performed the present meta-analysis. PubMed, EMBASE, and Web of Science database searches were performed before 1 April 2021 using the following terms: glutathione S-transferase, glutathione S-transferase pi, GST, *GSTP1*; genetic, polymorphism, variant; and myocardial infarction, MI, coronary heart disease, CHD, coronary artery disease, CAD, ischemic heart disease. The references of eligible articles and relevant reviews were also screened for additional reports. All identified publications should fulfill the following criteria: (a) designed as case-control studies, (b) assessed the association of *GSTP1* Ile105Valpolymorphism with CHD risk published in English, (c) provided the adequate genotypic information to calculate odds ratios (ORs) and 95% confidence intervals (95% CIs). Editorials, review articles, animal studies, case reports, preliminary results not on *GSTP1* gene polymorphism or CHD, and studies without data for estimating OR with 95% CI were excluded.

### Data extraction and quality assessment

For each included study, the following data were extracted and entered into the standard form: first author, year of publication, country, ethnicity, source of controls, number of cases, number of controls, G allele (%) in case, G allele (%) in control, Hardy-Weinberg equilibrium (the genotype distribution in controls were consistent with Hardy-Weinberg equilibrium (HWE)), Newcastle-Ottawa Scale (NOS) score, and Adjustment covariance. Because two publications [12,22] only provided the genotype data as “Val/Val+Val/Ile” and “Ile/Ile” without details, we could only estimate the OR for the dominant genetic model. We conducted the quality assessment of eligible publications based on NOS [2,26–28]. Two authors (C.L and L.C) independently performed the database searches, data extraction, and quality assessment of included studies, and any disagreements were discussed and resolved with a third investigator (X.L.L).

### Statistical analysis

The pooled ORs and 95% CIs were used to estimate the strength of the association between *GSTP1* Ile105Valpolymorphism and susceptibility to CHD. We investigated the association using five genetic models, including dominant model (Val/Val+Val/Ile vs. Ile/Ile), homozygote model (Val/Val vs. Ile/Ile), heterozygote model (Val/Ile vs. Ile/Ile), recessive model (Val/Val vs. Val/Ile + Ile/Ile), and allele model (Val vs. Ile). Both the Cochran Q test and *I*^*2*^ test were performed to evaluate the between-study heterogeneity among included studies [29]. Significant heterogeneity was detected when *P*<0.10 for the Q test or *I*^*2*^≥50% for *I*^*2*^ statistic, and a random effect model using DerSimonian-Laird method was applied. Otherwise, a fixed effect model using Mantel-Haenszel method was performed if heterogeneity was negligible [10]. The detection of outliers by Galbraith plots was considered as the main source of between-study heterogeneity [30]. Publication bias was assessed using Begg’s funnel plot and Egger’s test, and *P*<0.05 indicated a potential publication bias [31–33]. For the control group in each selected study, the Chi-square goodness-of-fit test was used to evaluate HWE and *P*<0.05 was considered significant. The meta-regression was performed with the ‘metareg’ STATA command to explore the source of between-study heterogeneity. Prespecified sources of heterogeneity included publication year, ethnicity, and control source. Sensitivity analysis, excluding one study at a time, was conducted to evaluate the stability of the results. Moreover, sensitivity analysis by excluding studies without confirmation of HWE was also performed. Stratified analyses were also performed by ethnicity (East Asian, Caucasian); source of controls (population-based, hospital-based). All analyses were performed with STATA (version11.0; Stata Corporation, College Station, TX).All tests presented are 2-tailedwith a significance level of 0.05.

## Results

### Identifying studies and study characteristics

The selection process of articles was presented in S1 Fig, with specification of reasons. 414 publications were found with the search criterion, and fifteen publications finally met the criteria for entering the present analysis. Because two articles [12,22] only reported data on genotypes as ‘‘Val/Val + Val/Ile” and ‘‘Ile/Ile”, the HWE test could not be conducted in these two studies and we could only estimate the OR for the dominant genetic model. The genotype distribution in controls was not in agreement with the HWE in one study [21]. The main study characteristics are summarized in Table 1 (Table 1). Finally, a total of 15 case-control studies with 5,451 CHD cases and 5,561 controls were included. There were eight studies on subjects of Caucasian and seven studies on subjects of Asia. The controls were divided into population-based population and hospital-based patients. The number of cases ranged from 54 to 2042, and the number of controls ranged from 78 to 2042. The mean distribution frequency of the *GSTP1* G allele was 31.28% in cases and the average frequency was 30.41% in controls.

**Table 1:** Characteristics of studies included in the meta-analysis.

First author	Year	Country	Ethnicity	Control source	Genotyping method	Cases	Controls	G allele (%)		HWE	NOS Score	Adjustment covariates
								Case	Control			
Wilson [4]	2000	UK	Caucasian	PB	PCR-RFLP	351	190	34.8	36.8	0.384	8	NA
Wang [12]	2007	Taiwan	East Asian	PB	PCR-RFLP	279	325	NA	NA	NA	7	Age and gender
Cornelis [8]	2007	Canada	Caucasian	PB	PCR-RFLP	2042	2042	39.3	39.4	0.646	8	Age, sex, area, smoking, waist-to-hip ratio, income, physical activity, history of diabetes and hypertension, intake of alcohol, and energy adjusted saturated fat and folate
Ramprasath [13]	2011	India	East Asian	PB	PCR-RFLP	290	270	39.3	32.2	0.093	6	NA
Singh [14]	2011	India	East Asian	PB	PCR-RFLP	230	300	20.9	22.8	0.08	7	Age, sex, BMI, smoking, alcohol, food habit, lipid profile and fasting glucose
Nomani [7]	2011	Iran	Caucasian	HB	PCR-RFLP	209	108	30.9	31.9	0.371	6	NA
Kariz [15]	2012	Slovenia	Caucasian	HB	PCR	206	257	37.9	32.9	0.731	5	NA
Phulukdaree [16]	2012	South Africa	East Asian	PB	PCR-RFLP	102	100	20.1	29.5	0.413	7	NA
Yeh [17]	2013	Taiwan	East Asian	HB	PCR-RFLP	458	209	15.9	16	0.176	5	Age, sex, cigarette smoking, alcohol use, diabetes mellitus, and levels of serum total cholesterol and high-density lipoprotein cholesterol
Kovacs [18]	2014	Hungary	Caucasian	HB	PCR	53	78	34.9	32.7	0.392	6	NA
Ding [19]	2016	USA	Caucasian	HB	PCR	119	382	27.7	32.5	0.268	7	Age, BMI, smoking status (ever/never) and total cholesterol to HDL cholesterol ratio
Bhat [20]	2017	India	East Asian	PB	PCR-RFLP	200	200	39	28.8	0.056	7	Age, gender, BMI, alcohol intake, total cholesterol, hypertension and family history
Bhatti [21]	2018	India	East Asian	PB	PCR-RFLP	560	545	38.2	32.1	0.0095	8	NA
Simeunovic [22]	2019	Serbia	Caucasian	PB	PCR-RFLP	107	274	NA	NA	NA	7	Gender, age, smoking, hypertension, and diabetes
Pourkeramati [23]	2020	Iran	Caucasian	PB	PCR-RFLP	244	281	26.6	27.76	0.277	7	Age and sex

NA = not available; PB = population based; HB = hospital based; HWE = Hardy-Weinberg Equilibrium; NOS = Newcastle–Ottawa Scale.

### Quantitative synthesis

**Fig 1:**
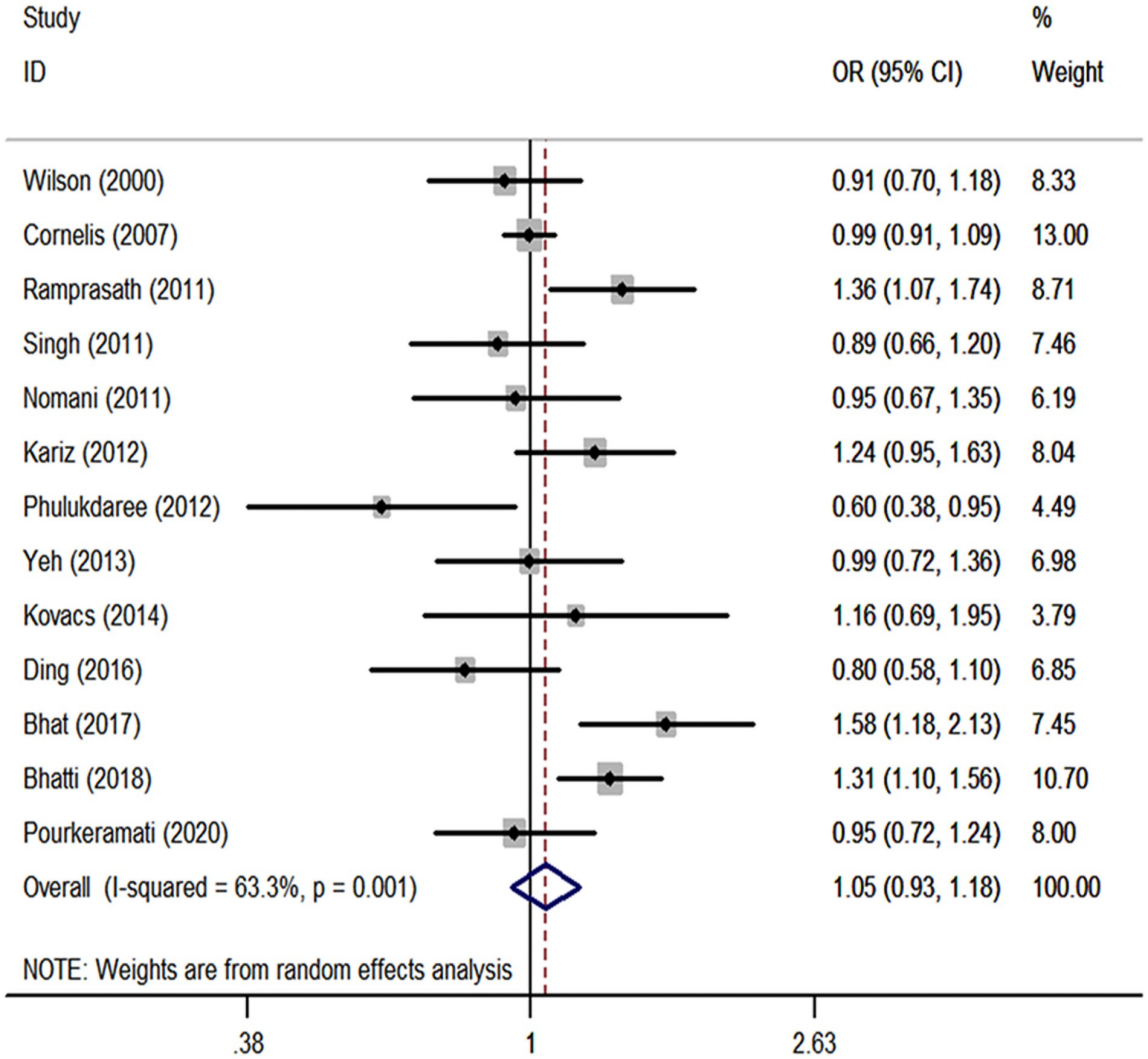
Forest plots for the *GSTP1* Ile105Val polymorphism and the risk of CHD in overall studies (Val vs. Ile).

**Fig 2:**
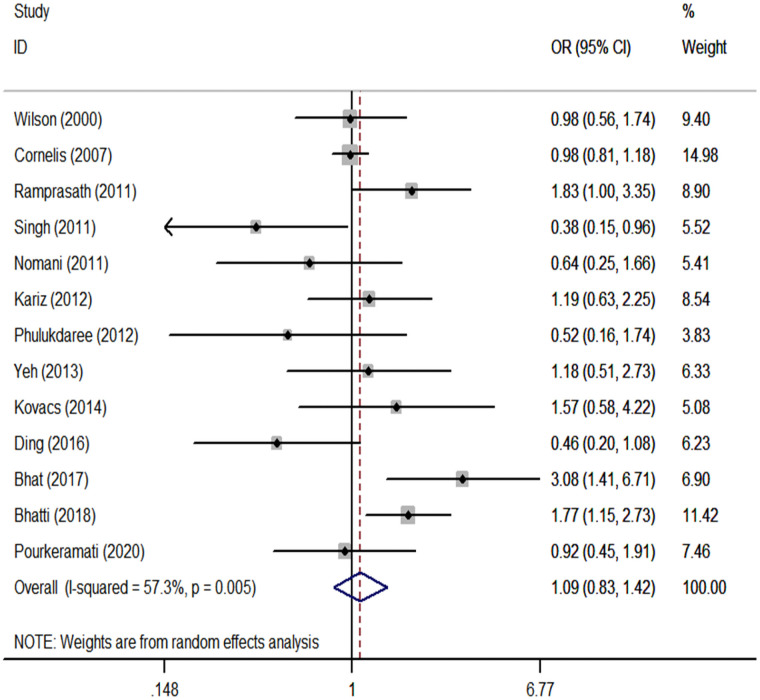
Forest plots for the *GSTP1* Ile105Val polymorphism and the risk of CHD in overall studies (Val/Val vs. Ile/Ile).

**Fig 3:**
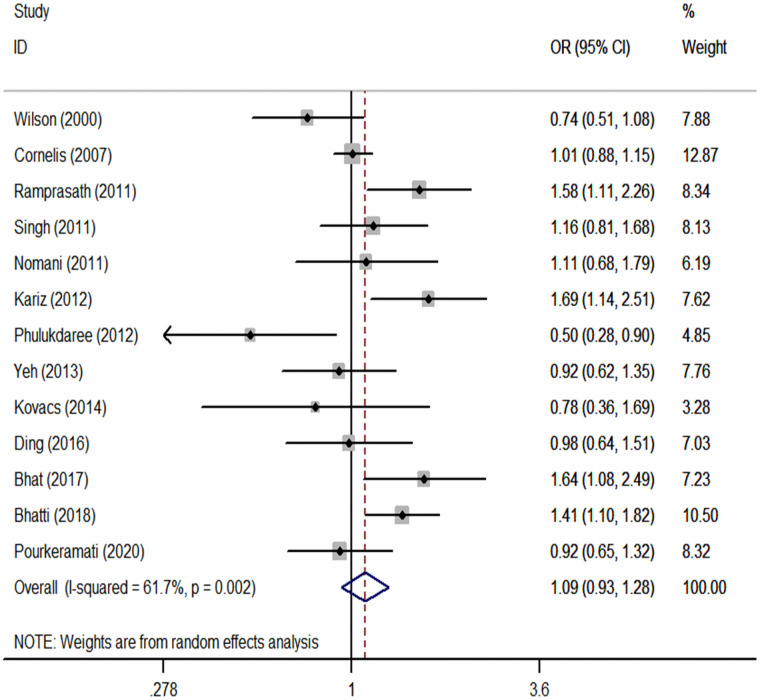
Forest plots for the *GSTP1* Ile105Val polymorphism and the risk of CHD in overall studies (Val/Ile vs. Ile/Ile).

**Fig 4:**
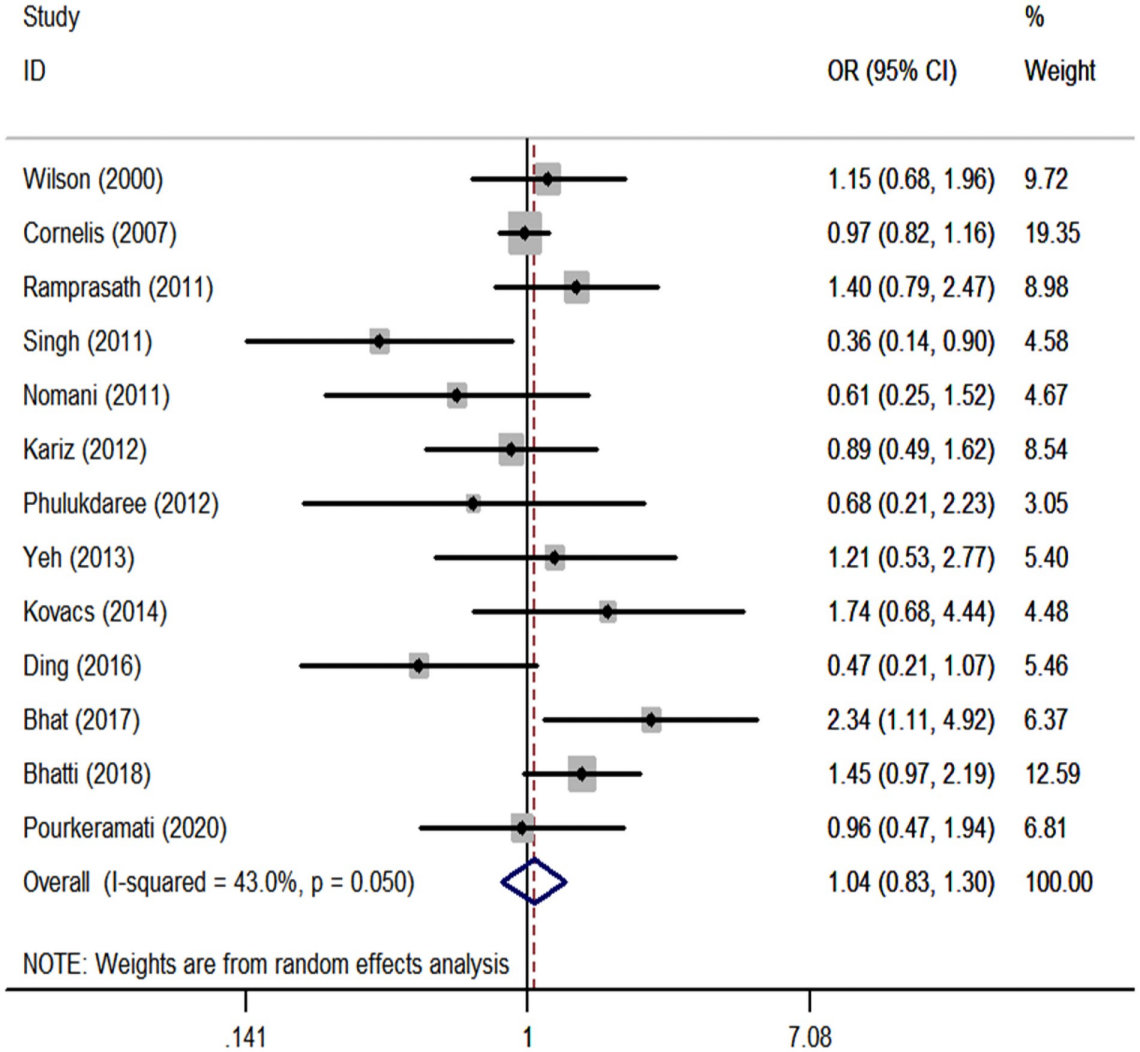
Forest plots for the *GSTP1* Ile105Val polymorphism and the risk of CHD in overall studies (Val/Val vs. Val/Ile+Ile/Ile).

**Fig 5:**
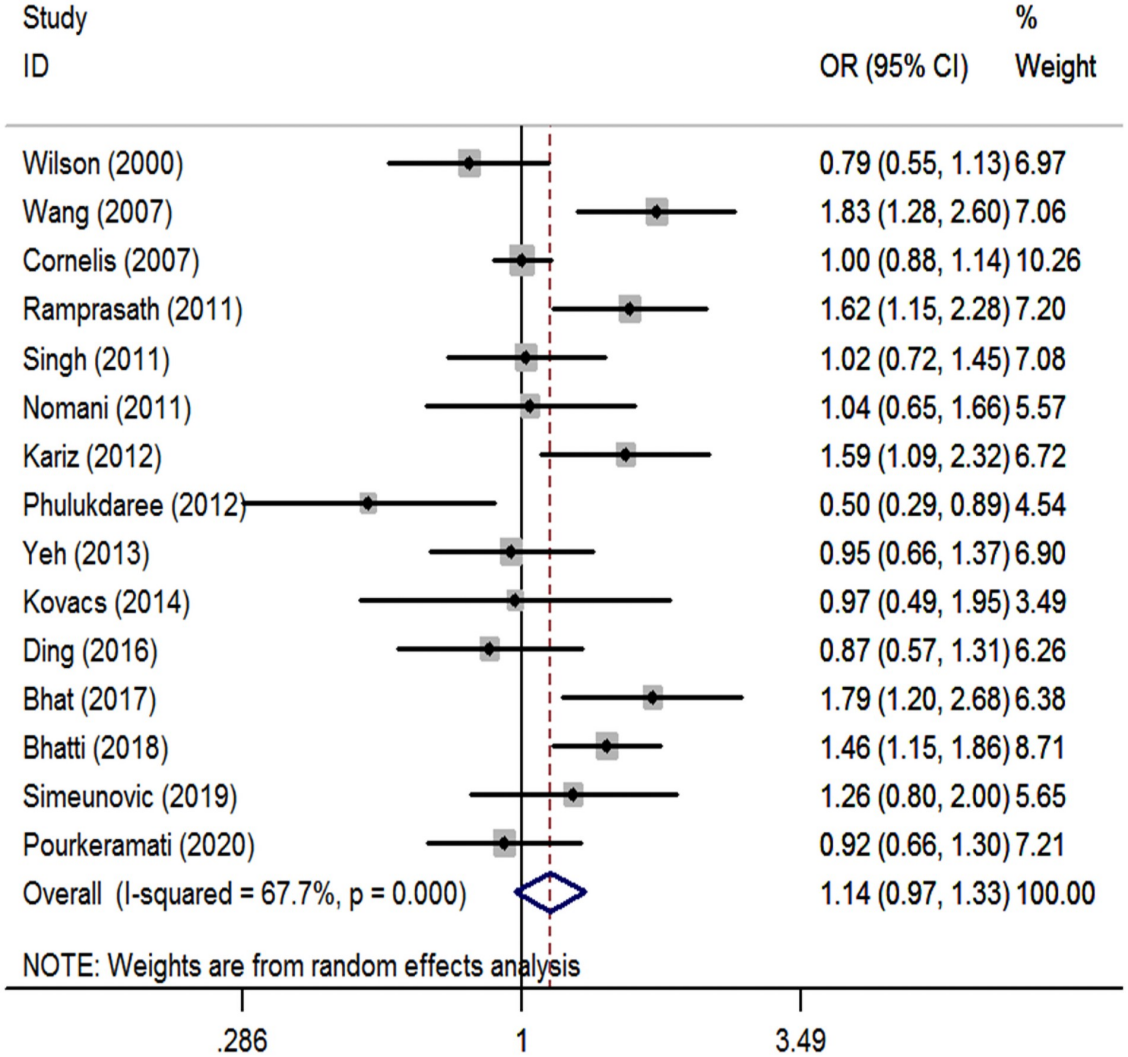
Forest plots for the *GSTP1* Ile105Val polymorphism and the risk of CHD in overall studies (Val/Val+Val/Ile vs. Ile/Ile).

We performed a meta-analysis of the *GSTP1* Ile105Val polymorphism under specific genetic models (Table 2). We found that *GSTP1* Ile105Val polymorphism was not associated with CHD risk for overall populations (Val vs. Ile: OR, 1.05; 95% CI, 0.93 to 1.18, Fig 1; Val/Val vs. Ile/Ile: OR, 1.09; 95% CI, 0.83 to 1.42, Fig 2; Val/Ile vs. Ile/Ile: OR, 1.09; 95% CI, 0.93 to 1.28, Fig 3; Val/Val vs. Val/Ile+Ile/Ile: OR, 1.04; 95% CI, 0.83 to 1.30, Fig 4; Val/Val+Val/Ile vs. Ile/Ile: OR, 1.14; 95% CI, 0.97 to 1.33, Fig 5). By stratifying the analysis by ethnicity, the present meta-analysis revealed that *GSTP1* Ile105Val polymorphism was not associated with CHD risk in Caucasian and East Asian (Table 2). In subgroup analysis according to the control source, *GSTP1* Ile105Val polymorphism was also not associated with CHD risk among hospital-based controls and healthy controls (Table 2). Covariates were introduced including the year of publication, ethnicity, and control source for meta-regression analysis. The meta-regression was conducted with the introduction of covariates including the publication year, ethnicity, and control source. However, in any comparison, no covariate was detected as a potential source of between-study heterogeneity.

**Table 2:** Summary ORs and 95% CIs of the association between *GSTP1* Ile105Val polymorphism and CHD risk.

Contrast model	Studies, n	Odds ratio	Heterogeneity		Model
		OR (95% CI)	*I*^*2*^	*P*_*H*_	
Total studies					
Val vs Ile	13	1.05 (0.93,1.18)	63.3%	0.001	Random
Val/Val vs Ile/Ile	13	1.09 (0.83,1.42)	57.3%	0.005	Random
Val/Ile vs Ile/Ile	13	1.09 (0.93,1.28)	61.7%	0.002	Random
Val/Valvs Val/Ile+Ile/Ile	13	1.04 (0.83,1.30)	43.0%	0.05	Random
Val/Val+Val/Ile vs Ile/Ile	15	1.14 (0.97,1.33)	67.7%	<0.001	Random
Caucasian					
Val vs Ile	7	0.99 (0.92,1.06)	0.0%	0.499	Fixed
Val/Val vs Ile/Ile	7	0.96 (0.82,1.12)	0.0%	0.551	Fixed
Val/Ile vs Ile/Ile	7	1.01 (0.91,1.13)	39.7%	0.127	Fixed
Val/Val vs Val/Ile+Ile/Ile	7	0.96 (0.83,1.11)	0.0%	0.431	Fixed
Val/Val+Val/Ile vs Ile/Ile	8	1.01 (0.92,1.12)	22.6%	0.25	Fixed
East Asian					
Val vs Ile	6	1.11 (0.89,1.39)	74.2%	0.002	Random
Val/Val vs Ile/Ile	6	1.27 (0.74,2.19)	68.8%	0.007	Random
Val/Ile vs Ile/Ile	6	1.18 (0.89,1.55)	68.9%	0.007	Random
Val/Val vs Val/Ile+Ile/Ile	6	1.17 (0.75,1.82)	56.50%	0.042	Random
Val/Val+Val/Ile vs Ile/Ile	7	1.26 (0.96,1.65)	74.80%	0.001	Random
PB					
Val vs Ile	8	1.07 (0.91,1.25)	74.8%	<0.001	Random
Val/Val vs Ile/Ile	8	1.16 (0.81,1.66)	68.8%	0.002	Random
Val/Ile vs Ile/Ile	8	1.09 (0.88,1.34)	71.8%	0.001	Random
Val/Val vs Val/Ile+Ile/Ile	8	1.11 (0.85,1.47)	52.0%	0.042	Random
Val/Val+Val/Ile vs Ile/Ile	10	1.16 (0.95,1.43)	76.1%	<0.001	Random
HB					
Val vs Ile	5	1.02 (0.88,1.18)	15.2%	0.318	Fixed
Val/Val vs Ile/Ile	5	0.92 (0.65,1.32)	22.0%	0.274	Fixed
Val/Ile vs Ile/Ile	5	1.12 (0.92,1.37)	37.8%	0.169	Fixed
Val/Val vs Val/Ile+Ile/Ile	5	0.85 (0.61,1.20)	26.8%	0.243	Fixed
Val/Val+Val/Ile vs Ile/Ile	5	1.08 (0.90,1.31)	29.4%	0.226	Fixed

PB = population based; HB = hospital based; 95% CI = 95% Confidence Interval; *P*_*H*_ = *P* value based on Q test for between-study heterogeneity.

### Heterogeneity and sensitivity analysis

Heterogeneity was detected in the overall pooled analysis (Table 2). After excluding studies detected by Galbraith plot as major sources of heterogeneity, no evidence for heterogeneity was detected and the fixed effects summary estimate also indicated that *GSTP1* Ile105Val polymorphism was not associated with an increased risk of CHD (Val vs. Ile: OR, 0.98; 95% CI, 0.91 to 1.05; *I*^*2*^ = 27.7%, *P*_*H*_ = 0.256, excluding 4 studies [13,16,20,21]; Val/Val vs. Ile/Ile: OR, 0.99; 95% CI, 0.86 to 1.16; *I*^*2*^ = 12.0%, *P*_*H*_ = 0.333, excluding 3 studies [14,20,21]; Val/Ile vs. Ile/Ile: OR, 1.01; 95% CI, 0.92 to 1.12, *I*^*2*^ = 14.5%, *P*_*H*_ = 0.313, excluding 4 studies [13,15,16,21]; Val/Val vs. Val/Ile+Ile/Ile: OR, 1.04; 95% CI, 0.88 to 1.23; *I*^*2*^ = 11.1%, *P*_*H*_ = 0.339, excluding 2 studies [14,20]; Val/Val+Val/Ile vs. Ile/Ile: OR, 1.01; 95% CI, 0.92 to 1.11; *I*^*2*^ = 1.5%, *P*_*H*_ = 0.424, excluding 5 studies [12,13,16,20,21]).

Sensitivity analyses were performed by omitting each study at a time to explore the effect of individual study, and the pooled ORs were not noticeably changed, suggesting that the results of present analysis were stable. In the sensitivity analysis, the influence of each study on the pooled OR was examined by repeating the meta-analysis while omitting each study, one at a time. This procedure confirmed the stability of the overall result. After omitting one study [21] departing from HWE and two studies [12,22] lacking the necessary information, the results based on crude ORs remained unchanged.

### Publication bias

Begg’s and Egger’s tests were performed to evaluate publication bias in the overall pooled analysis. Begg’s and Egger’s test indicated no significant evidence of publication bias (Val vs. Ile: Begg’s test, *P* = 0.428; Egger’s test, *P* = 0.965; Val/Val vs. Ile/Ile: Begg’s test, *P* = 0.300; Egger’s test, *P* = 0.967; Val/Ile vs. Ile/Ile: Begg’s test, *P* = 0.583; Egger’s test, *P* = 0.934; Val/Val vs. Val/Ile+Ile/Ile: Begg’s test, *P* = 0.360; Egger’s test, *P* = 0.882; Val/Val+Val/Ile vs. Ile/Ile: Begg’s test, *P* = 0.767; Egger’s test, *P* = 0.766).

## Discussion

In the present meta-analysis including 15 studies with a total of 5,451 CHD cases and 5,561 controls, the association between *GSTP1* Ile105Val polymorphism and CHD risk was comprehensively assessed, and no positive results were obtained by the overall analysis.

Recently, one previous meta-analysis, conducted by Su et al. [24], included 4,595 cases and 4,390 controls from 11 studies. They proved that *GSTP1* null polymorphism was associated with the risk of CHD in the overall population (OR, 1.23; 95% CI, 1.02 to 1.48). Another meta-analysis performed by Li et al. [25] revealed that *GSTP1* null genotype could impact individual susceptibility to atherosclerotic cardiovascular diseases. However, our meta-analysis indicated that the *GSTP1* Ile105Val polymorphism was not associated with CHD risk in five genetic models. The present study included 5,451 CHD cases and 5,561 controls in 15 studies, which could provide more sufficient statistical power. Compared with previous studies, more than four relevant studies [4,19,22,23] were involved in present meta-analysis but not in theirs. The sample size of two previous studies was relatively small and data was not sufficient for subgroup analysis. The results were not changed after adjustment for heterogeneity by excluding studies spotted by Galbraith plot. When stratifying the analysis by ethnicity, the results of our study revealed that *GSTP1* Ile105Val polymorphism was also not associated with CHD risk in East Asian and in Caucasian.

The evaluation of heterogeneity is critical to the interpretation of the results for most meta-analyses [34]. Even modest heterogeneity exists across eligible studies may make meta-analysis miss the true effect. In the present meta-analysis, obvious between-study heterogeneity existed in five genetic models for overall populations. The heterogeneity persisted when stratified analyses were conducted by ethnicity and control source. Furthermore, a Galbraith plot was conducted to explore the source of heterogeneity. After excluding studies with low-quality design, no obvious between-study heterogeneity was observed among the remaining studies. In addition, sensitivity analysis was performed to confirm the robustness of our findings. The between-study heterogeneity may be caused by the inclusion population or study design. For example, Nomani et al. [7], Ramprasath et al. [13] and Cornelis et al. [8] contained a high risk control group with hypertension, diabetes mellitus, or family history of CHD. The results may be biased when the controls can not reflect exposure distribution or the genotype of the source population.

It was reported that *GSTP1* Ile105Val polymorphism was associated with the altered catalytic and non-catalytic activity of GSTs. The GSTP1*G allele coding for the protein in which amino acid isoleucine (Ile) is substituted with valine (Val) has been shown to reduce enzyme activity and affinity for electrophilic substrates, which may lead to individual susceptibility to CHD. Of the fifteen studies, eight reported no significant association between *GSTP1* Ile105Val polymorphism and the risk of CHD. Among the rest of the studies, Wang et al. [12] observed a 1.8-fold increased CHD risk among subjects with the combination of Val/Val and Val/Ile genotypes of *GSTP1* when compared to Ile/Ile genotype. Ramprasath et al. [13] found that *GSTP1* Ile105Val polymorphism was associated with a higher risk of CHD. Singh et al. [14] reported that the interactive effect of *GSTP1* Val/Val with MI remained significant after adjusting for risk factors. Kariz et al. [15] proved that univariate analysis indicated an association between the *GSTP1* Ile105Val polymorphism and MI. Phulukdaree et al. [16] found that a significant association with CHD was observed in *GSTP1* A105/A105. Bhat et al. [20] reported a statistically significant association between *GSTP1* g.313A>G (A/G, G/G) genotype and CHD was detected. The study by Bhatti et al. [21] indicated the GG genotype of the *GSTP1* (313A/G) gene was associated with an approximately two-fold enhanced risk of developing CHD. However, when pooling all studies together, we found no evidence for an association between *GSTP1* Ile105Val polymorphism and CHD. The etiological mechanism of CHD is very complicated, in which gene-gene and gene-environment interactions may play important roles. The findings of the study by Phulukdaree et al. [16] supported the association of genotypes *GSTM1* 0/0 and *GSTP1* A105/A105 and smoking with CHD. The study by Simeunovic et al. [22] observed a stronger association in heart failure patients due to CHD, who were carriers of a combined *GSTP1*(rs1695)/*GSTA1*“risk-associated” genotype. Singh et al. [14] reported that a significant protective effect of *GSTP1* Val genotype against the disease was detected in nonsmokers with AMI. Hence, future studies concerning gene-gene, gene-nutrition, and gene-environment interactions under a systems network biology framework are required [35–39]. N4-acetylcytidine (ac4C) is often known as a conservative, chemically modified nucleoside present on rRNA and tRNA. The abnormal expressions of some gene indications are mediated through mRNA modifications. The recent progress of N4-Acetylcytidine on RNA expression plays a very important part in human diseases [40]. Future studies should be conducted to elucidate the potential biological regulation mechanisms regarding how the genetic variant affect the CHD outcome through N4-Acetylcytidine on RNA expression.

Some limitations of the present meta-analysis should be highlighted. First, our meta-analyses were based on unadjusted estimates. Future studies with potential confounding factors, such as age, ethnicity, sex, lifestyle factors and environmental exposure factors, should be conducted if possible. Second, obvious heterogeneity was detected in this study. We have performed meta-regression, Galbraith plots, sensitivity analysis, and subgroup analysis and the results of the present meta-analysis did not change. Third, the number of including publications and the sample size were relatively small. So, the findings should be interpreted with caution. Four, only one SNP within *GSTP1* is not enough to elucidate the role of this gene on the susceptibility to CHD. Genome-wide association studies in the future should be conducted to investigate the association between single nucleotide polymorphisms (SNPs) in the *GSTP1* gene and the risk of CHD [41]. Future studies should also be performed to see if the *GSTP1* Ile105Val polymorphism or other SNPs in this gene are causally triggering the development of CHD through mediating the expression of this gene in specific tissues, like vascular or heart [42–44]. Finally, gene-gene, gene-nutrition, and gene-environment interactions were not performed for the lack of sufficient information. Deep learning or machine learning is a hot topic in the classification and prediction of diseases based on biomarkers, and future studies should concentrate on the genetic variants, gene-gene, and gene-environment interactions for the prediction or early diagnosis of CHD [45,46].

## Conclusions

In conclusion, our meta-analysis indicated that *GSTP1* Ile105Val polymorphism did not appear to confer susceptibility to CHD. Further well-designed studies with detailed personal information are needed to validate the results of the present study.

## Supporting information

S1 Checklist PRISMA-P 2015 checklist. (DOCX)

S1 Fig PRISMA flowchart describing the included/excluded literature. (TIF)
