# Supplementary figures and images for: Association of GSTP1 Ile105Val polymorphism with the risk of coronary heart disease: An updated meta-analysis
Source: PLoS One. 2021 Jul 22;16(7):e0254738. doi: 10.1371/journal.pone.0254738 (PMC8297824; doi:10.1371/journal.pone.0254738)

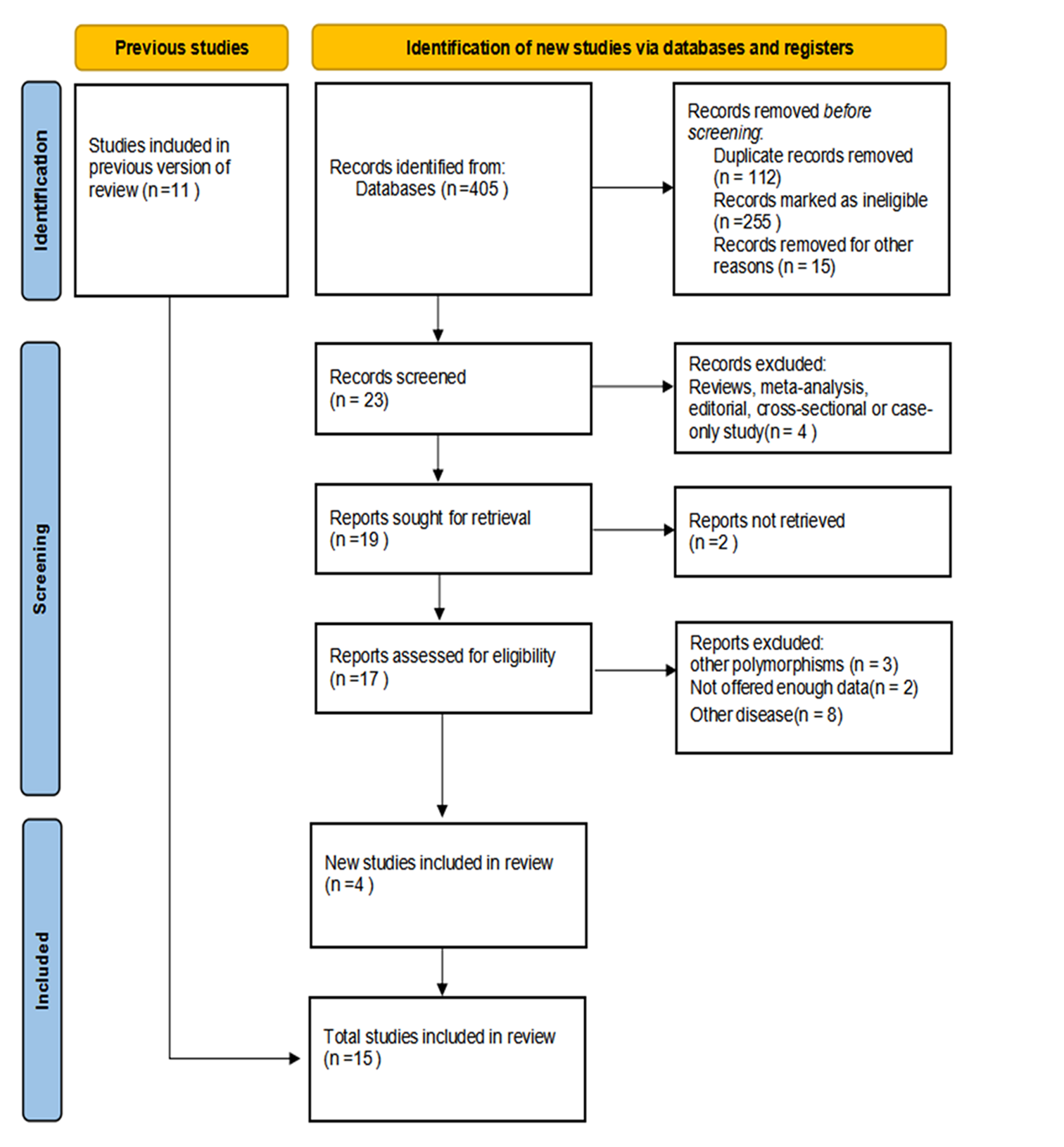

Supplement: S1 Fig — (TIF) [file pone.0254738.s002.tif]
